# Psychological Interventions for Dementia Caregivers: What We Have Achieved, What We Have Learned

**DOI:** 10.1007/s11920-019-1045-9

**Published:** 2019-06-06

**Authors:** Sheung-Tak Cheng, Alma Au, Andrés Losada, Larry W. Thompson, Dolores Gallagher-Thompson

**Affiliations:** 1 Department of Health and Physical Education, The Education University of Hong Kong, Tai Po, Hong Kong; 2 Department of Clinical Psychology, Norwich Medical School, University of East Anglia, Norwich Research Park, Norwich, NR4 7TJ UK; 3 Department of Applied Social Sciences, The Hong Kong Polytechnic University, Hung Hom, Hong Kong; 4 Psychology Department, Universidad Rey Juan Carlos, Madrid, Spain; 5 Department of Psychiatry and Behavioral Sciences, Stanford University School of Medicine, Stanford, CA 94305 USA; 6 Betty Irene Moore School of Nursing/Family Caregiving Institute, University of California, Davis, CA 95616 USA

**Keywords:** Dementia, Alzheimer’s disease, Caregiver interventions, Cross-cultural issues

## Abstract

With the rising dementia population, more and more programs have been developed to help caregivers deal with the care-recipient as well as their own frustrations. Many interventions aim to enhance caregiver’s ability to manage behavior problems and other deteriorations in functioning, with less direct emphasis placed on caring for the caregivers. We argue that techniques based on psychotherapy are strategically important in assistance provided to caregivers because of their utility for promoting emotional health. This article provides a focused review of such methods used in evidence-based intervention programs, along with the mechanisms of change associated with these methods. While cognitive-behavioral therapy (CBT) has a strong evidence base, there is also a growing trend to package CBT techniques into various psychoeducational programs. These programs, which we call psychoeducation with psychotherapeutic programs, have been consistently found to be effective in reducing caregiver distress and are suited for delivery in group format, even by paraprofessionals, to lower the cost of intervention. A recent trend is the effective use of technological aids (e.g., the internet) to deliver CBT and psychoeducation, reaching more caregivers. As for therapeutic mechanisms, the use of coping skills, reduced dysfunctional thoughts, and increased self-efficacy in controlling upsetting thoughts has received support in studies. We conclude that psychotherapeutic techniques are increasingly being used effectively and efficiently to assist caregivers, aided by successful adaptation for educational or technologically advanced means of delivery. More research on therapeutic mechanisms is needed to understand how the techniques work and how they can be further refined.

## Introduction

A substantial proportion of the dementia care costs (40% in high-income countries and 70–90% in low-to-middle-income countries) are accounted for by the work of informal caregivers (mostly family members and occasionally friends and neighbors) who provide unpaid care on a nearly round-the-clock basis for many years [1]. On top of the daily activities, dementia caregivers need to handle neuropsychiatric symptoms that are unpredictable, disturbing, sleep-depriving, and potentially embarrassing and abusive. Among the neuropsychiatric symptoms, disruptive behaviors (e.g., agitation, aggression, and sexual disinhibition) are the most distressing and challenging because of the potential harm to the caregiver’s bonding with the care-recipient (CR) and partly because they exacerbate difficulties in other domains (e.g., caring for activities of daily living). Besides the disruptive behaviors, delusions (e.g., delusion of infidelity) and mood disturbance are also quite challenging for caregivers [2]. These issues, together with other difficulties (e.g., coping with multiple roles, family conflicts) generate chronic stress and exhaustion that put most caregivers at risk for various physical and psychological morbidities, including cardiovascular diseases, depression, and anxiety [2].

Clearly, there is a need to improve our support for informal caregivers [3], especially in view of the impending dementia pandemic [1]. For this reason, a critical review of effective interventions for caregivers, as well as ways to improve research and practice, is provided here. Unlike previously published reviews [4–6], this article focuses on the psychological ingredients in many effective caregiver interventions that are based on established methods in the practice of psychiatry and psychology. Where data are available, we will also discuss the psychological mechanisms of change associated with these programs. Also covered are new ways (aided by technology) by which these interventions are reaching more caregivers than before. The need to address cultural and language barriers in multiethnic communities will also be discussed.

On top of a brief overview of the historical development of these interventions, we will focus on randomized controlled trials (RCTs) in the past 5–6 years, although translational programs based on evidence-based interventions will also be covered in the review. Readers interested in earlier intervention studies should consult two former reviews [7, 8]. Before we proceed to this part, an articulation of theoretical rationales as to why psychological methods are essential to dementia caregiver interventions is warranted.

## Psychological Models of Caregiver Stress

The stress process model [9] which depicts caregiver outcomes as determined by primary (e.g., care demands) and secondary stressors (e.g., role conflicts, financial strain), with mediators such as coping and social support, has dominated the field. Significant proportion of the variance in caregiver distress has been explained by employing this model [10, 11]. However, caregiver outcomes depend not only on objective factors such as the presence of neuropsychiatric symptoms, care hours and financial problems but also on subjective appraisal [12]. For instance, many caregivers think that the problem behaviors are under the CR’s control [13], who would likely be more upset than others who attribute the behaviors to the disease. On the contrary, caregivers with certain frames of mind are more inclined than others to find positive gains despite hardship, such as a sense of purpose, increased closeness with the CR, and feelings of mastery and gratification [14, 15]. These appraisals may be related to cognitive vulnerabilities such as dysfunctional thoughts [16, 17]. Further supporting the cognitive model, research has suggested that self-efficacy in controlling upsetting thoughts, rather than self-efficacy in managing disruptive behaviors or in obtaining respite, is an important predictor of caregiver outcomes, including being a primary mechanism of change in psychological interventions based on the cognitive model [18–21].

Research has identified other psychological models that are relevant for explaining caregiver stress [22]. Drawing upon the sociocultural stress-and-coping model [23], Losada and colleagues [16] showed the connection between cultural values and caregiving dysfunctional thoughts. For example, traditional beliefs about family obligations in Asian and Latino/Hispanic communities may lead to dysfunctional thoughts emphasizing the need for complete dedication to caregiving at the expense of one’s own needs and feelings. Avoidant coping may ensue when caregivers feel pressurized to do it regardless of personal choice [24].

More recently, studies carried out in the context of third-wave cognitive-behavioral therapies suggest the need to take into consideration variables such as experiential avoidance [25] and commitment to values [26]. Fostering acceptance of aversive experiences and commitment to personal values is particularly relevant in the context of the chronic and intractable nature of the challenges and experiences including sadness and grief. Furthermore, even though there is usually one member of the family that takes the lead role of caregiving duties, caregiving impacts the whole family, and so it is not surprising that systemic and interpersonal models are also useful [27]. Finally, studies based on behavioral models have shown the importance of increasing pleasant activities engaged by caregivers (i.e., behavioral activation and activity restriction) [28–30].

These are some examples of psychological approaches that complement the stress process model in explaining caregiver distress. Many interventions were based on these psychological models, whether implicitly or explicitly. However, with studies’ usual primary aim to evaluate the efficacy or effectiveness of interventions, the underlying theoretical constructs mentioned above were seldom assessed as intermediate or secondary outcome variables, thus hindering theoretical development. With this limitation of the literature in mind, we now provide a critical review of studies to illustrate how psychological models have influenced the development of interventions for caregivers.

## Effectiveness of Psychological Interventions for Dementia Family Caregivers

The past few years have seen tremendous expansion in the number and quality of studies designed to improve well-being (variously defined) in dementia family caregivers, both in the USA and internationally. In this review, we focus on four types of caregiver interventions that have psychological ingredients, namely psychoeducation, counseling and psychotherapy, multicomponent interventions (only those including psychological methods), and mindfulness-based interventions (Table 1). Although outcome measures and methodological rigor vary, for the studies included in this review, effect sizes are generally moderate-to-large for depressive symptoms, and small-to-moderate for anxiety, perceived burden, and overall quality of life measures in RCTs. We focus on studies reporting these outcomes because of their relevance to psychological interventions. Besides, we will also highlight interventions utilizing technologies and gadgets (e.g., DVD, telephone, videophone, internet, pocket-PC) to deliver non-face-to-face or self-administered interventions. Although these might be psychoeducation, psychotherapy, or multicomponent programs, we will discuss them together to highlight the growing efforts to reach and engage more caregivers with minimal disruptions of their daily routine, and to address access issues in some communities. In the interest of space, we will not discuss the group of dyadic interventions.

**Table 1:** Types of psychological interventions for dementia caregivers

Psychoeducation	
Caregivers are taught adaptive skills for coping with caregiving demands and stress using a structured format and is often delivered in small groups including time for didactics and practice. The topics covered usually includes information about dementia and community services, learning to take time for self, improving communication with family, and skills for managing problem behaviors. More specialized skills such as anger management, thought modification, and pleasant event scheduling may also be covered	
Counseling and psychotherapy	
Individual and family counseling/psychotherapy is provided by trained providers to help caregivers manage stress and for the treatment of distress such as depression. This modality is often used for caregivers with clinical depression or other significant mental health problems.	
Multicomponent interventions	
These interventions consist of several established intervention strategies blended together in order to address a variety of caregiver needs. Ideally, the different strategies should be integrated in a fashion to enhance treatment efficacy. Education, skills training, counseling, problem-solving, support group, home modification, and “health passport” are some of the components that may be included	
Mindfulness-based interventions	
Caregivers are trained in mindfulness (mostly Mindfulness-Based Stress Reduction) or other meditation strategies with the basic aim of paying attention to the present momentary experience on purpose and nonjudgmentally. Experiences (thoughts, emotions, behaviors, etc.) are observed without being judged as good or bad with a final aim of relieving suffering	

### Psychoeducation

Development and evaluation of psychoeducational programs began about 20 years ago with the Coping With Caregiving (CWC) program of Gallagher-Thompson and colleagues [31]. Early versions focused on teaching caregivers how to manage problem behaviors (e.g., identifying what triggers the behavior), modify dysfunctional thoughts, increase pleasurable activities, and learn other skills for emotion regulation. These programs typically engage groups of 6–10 participants in which relevant information and procedures for a variety of techniques are presented; this is then followed by rehearsal and practice on how to apply techniques in the course of carrying out caregiving activities at home. The CBT content distinguishes this program from many other psychoeducational programs which emphasize more on providing information, support, basic coping strategies, and help-seeking [32–34], including the widely disseminated Savvy Caregiving program [35].

A caveat needs to be mentioned. By packaging CBT techniques into a psychoeducational format, the techniques are delivered on a module basis to a small group of caregivers in a standardized way, rather than on a more personal level and utilizing a therapeutic relationship, as in individual or group psychotherapy. The emphasis is on instruction of CBT concepts and skills to facilitate self-help in terms of coping with the ongoing challenges in caregiving. By doing so, trained paraprofessionals (including graduate students with appropriate backgrounds), rather than professional therapists, were often employed to be trainers. This approach has been demonstrated to be especially helpful when delivering interventions in minority communities [36] or other communities [19, 20, 29, 30] where professional therapists are lacking.

After initial success in pilot studies [31], several RCTs based on the CWC program were conducted with Caucasians, Asian-Americans (specifically Chinese and Japanese), Hispanic/Latino-Americans [8], and Spanish [37] Mexican caregivers [38] with considerable success. START (STrAtegies for RelaTives), in particular, is a program based on the CWC that has been rolled out in many parts of UK after demonstrating long-term effectiveness (up to 2 years) on depressive and anxiety symptoms [39, 40]. A specialized CWC-based program in anger management was also developed [18] that has been widely used by community-based programs in the state of California, USA.

More recently, other innovative psychoeducation programs have been reported. Garand and colleagues [41] adapted problem-solving therapy into a psychoeducational format, producing significant treatment effects on depressive and anxiety symptoms, as well as negative orientation toward problems. Gonzalez and colleagues [42] also evaluated a program based on problem-solving therapy and found treatment effect on anxiety but not depressive symptoms. Barnes and Markham [43] integrated CBT and communication skills training in a brief intervention, the Talking Sense program, but did not find any effect on depressive and anxiety symptoms, though the intervention group reported higher communication self-efficacy than control. A novel approach was described recently by Cheng and colleagues [19, 20, 44–47] who found that a benefit-finding intervention incorporating positive psychology principles and thought-modification (via promoting alternative thinking) had medium to large effect sizes for reducing depressive symptoms and subjective burden when compared with standard psychoeducation in Hong Kong Chinese caregivers.

### Counseling and Psychotherapy

The category of psychotherapy, especially CBT and associated behavioral approaches to treat depression, has grown substantially, particularly on the international scene. Moore and colleagues found that behavioral activation reduced, at posttreatment, depressive symptoms, negative affect, and an inflammatory marker (IL-6) in caregivers, compared with those receiving information-support control. However, the effects disappeared at 1-year follow-up [48]. This was one of the few trials ever to include biological outcomes. In a relatively large trial in the Netherlands, Joling and colleagues [49] tested the family counseling component from the NYU Caregiver Intervention Program but did not find effect on any outcome.

In Spain, Losada and colleagues [50] followed up on their earlier work on the efficacy of CBT to treat caregiver depression by conducting an RCT comparing CBT with acceptance and commitment therapy (ACT), a relatively unexplored form of treatment for caregivers. ACT does not emphasize challenging and changing unhelpful thoughts but rather accepting and adapting to what is. This can be a powerful message for caregivers who need to realize that decline is inevitable and there is only so much they can do for their impaired relative. Results indicated that both forms of therapy were equally effective to significantly reduce depression and anxiety. More research on ACT is needed to more firmly establish its efficacy for caregivers.

A culturally sensitive group CBT program (both face-to-face group sessions and telephone coaching were used) was developed for Latino Americans in US and was found to have effects on caregivers’ depressive symptoms, distress in relation to neuropsychiatric symptoms, and self-efficacy; caregivers also reported lower NPS in their CRs [51]. In Latin America, a group CBT was found to be more effective than an educational control to reduce depressive symptoms and perceived burden both at posttest and follow-up [52]. In Spain, Rodriguez-Sanchez and colleagues [53] also conducted group CBT but in a sample of mixed caregivers (i.e., not only dementia caregivers) in a primary care setting and found CBT superior to usual care in overall mental health improvement and in reducing dysfunctional thoughts about caregiving. In short, CBT appears to have a strong evidence base while more studies of ACT are recommended.

### Multicomponent Programs

Only multicomponent interventions that include psychoeducation and/or counseling are considered in this review. An exemplary program is the Resources for Enhancing Alzheimer’s Caregiver Health (REACH) II project that included over 600 caregivers from five sites in the US, with approximately equal numbers of Caucasian, African American, and Hispanic/Latino caregivers [54]. This program consisted of nine in-home sessions (90 min each) and three telephone sessions (30 min each). At post-evaluation, Latinos, whether spouse or adult child caregivers, made the greatest improvement on an omnibus measure of well-being, followed by Caucasians. African Americans had less overall improvement; in fact, recent reanalysis of this RCT by Graham-Phillips and colleagues [55] found that they actually completed fewer in-home sessions then and so, had less contact time. It is not clear whether certain components of the intervention had less appeal to this population. Recently, an abridged version of REACH II was adapted to German communities, with favorable results for burden and distress associated with challenging behaviors; the frequency of CRs’ challenging behaviors was also reported to have decreased, in comparison with usual care [56]. Furthermore, a brief and adapted version of REACH II was developed and disseminated in Spanish and English in southern San Diego county (CA, US) for the past 5 years. Results indicated significant improvement in depressive symptoms from pre- to post-intervention [57]. This intervention is noteworthy since materials were culturally tailored for Latino-American caregivers with low literacy.

Gaugler and colleagues’ work [58, 59], based on the NYU Caregiver Intervention program, can be considered multicomponent as several approaches were used. Originally, it consisted of two sessions of individual counseling with the spouse caregiver alone and four sessions of family counseling with caregiver and other family members, conducted by trained social workers. There was no predetermined end to treatment: Caregivers could request assistance (including additional counseling) at any time and many did. Similar to REACH, this was an expensive and time-consuming program, and so subsequent studies, while keeping counseling as the cornerstone of treatment, varied the contents and number of sessions. For example, Gaugler and colleagues [58] delivered this program to a small sample of adult child caregivers studied for 3 years and found significant decreases in depressive symptoms compared with minimal contact control. However, the average number of family counseling sessions utilized averaged only one, with nearly half of the caregivers not using any such sessions at all [59]. This program is being extended to Hispanic caregivers in New York City [60], although results are not yet available.

### Mindfulness-Based Interventions

Recent years have seen a growing interest in the effects of mindfulness-based interventions (MBI). Most of the studies focused on the Mindfulness-Based Stress Reduction (MBSR) intervention alone [61, 62] or combined it with Mindfulness-Based Cognitive Therapy (MBCT) [63]. Other studies have tested the effects of a combination of mindfulness mediation, breath-focused imagery and mantra repetition [64], transcendental meditation [65], or a combination of yoga and compassion meditation [66]. Whitebird and colleagues found positive results for the two interventions tested (MBSR vs. a community caregiver education and support), although the MBSR seemed to be more effective in improving mental health, stress, and depressive symptomatology [62]. While positive effects in psychosocial measures were also obtained by Brown et al. using the MBSR [61], Oken et al. did not find significant effects of MBSR and MBCT combined for most of the assessed variables, including mindfulness measures. Danucalov and colleagues reported significant improvements in stress and anxious and depressive symptomatology using a combination of yoga and compassion mediation intervention [66]. However, no effects on mental health outcomes or self-efficacy were obtained in two RCTs using different variants of meditation training [64, 65]. In studies with cortisol as outcome, mixed findings were reported. While Waelde et al. reported improvements in diurnal cortisol slope [64], no changes over time in cortisol response were observed by Brown et al. [61]. We note that the existing studies are small in number and have been done with small samples. There are also insufficient data concerning diverse and long-term outcomes. Methodological improvements are needed before strong conclusions about the efficacy of this type of interventions can be made.

### Technological Aids in Delivery

Face-to-face interventions are not always feasible because of the lack of time on the part of the caregiver due to the demanding nature of dementia caregiving or conflicts with other duties. Caregivers may also find it difficult to leave the CR to someone else in order to attend intervention meetings. Furthermore, services for caregivers are simply unavailable or difficult to access in many communities around the world, especially rural or under-developed communities.

In recent years, various forms of technology are being used more and more to deliver interventions and evidence is accumulating that these can be cost-effective ways to assist caregivers. For example, psychoeducational programs have been delivered successfully over the telephone [67, 68] or e-learning platforms [69]. CBT [70–73] and multicomponent programs [74] have also been conducted over the telephone or the internet with success. A novel effort to reach under-served Hispani/Latino caregivers in the US is the use of Fotonovela, a culturally accepted way to impart health information, to show how a Latino family copes adaptively with a dementia diagnosis (written in both Spanish and English). In the first RCT with > 100 Latino caregivers, results indicated improvement in depressive symptoms and caregiving-related stress over an information-only control [75].

Boots and colleagues [76] completed a systematic review of 12 internet-based intervention studies and concluded that available programs can improve aspects of caregiver well-being (such as confidence and self-efficacy) provided that they are tailored to the individual. They also noted that programs including interaction with a coach and/or other caregivers increased learning and social support and had better outcomes, though few were RCTs at the time. Exceptions included two innovative programs in Germany which delivered CBT over the telephone with excellent results. Wilz and colleagues [77] found both short- and long-term benefits (reduced depressive and physical symptoms, and improved mood and coping) for the CBT condition over progressive muscle relaxation or untreated control. Recently, the utility of CBT for managing loss and grief was evaluated. Meichsner and colleagues [71] developed a manualized telephone-CBT program in Germany that provided 12 50-min individual therapy sessions over 6 months. Compared with control, the program showed small but significant reductions in caregiver grief at post-intervention and 6-month follow-up. Using content analysis from the RCT to learn what themes were frequent and how CBT could help, they found that normalization of grief and redefinition of the relationship were key and cognitive reframing was often used [78].

A few web-based psychoeducation and multicomponent programs have also received research support. For instance, the Mastery over Dementia program was successfully evaluated in the Netherlands, with caregivers in the treatment condition reporting significantly less anxiety and depressive symptoms, compared with those in the education-only control. This program also included delivering CBT over the internet [72]. An adaptation of REACH II, delivered via a videophone, also showed positive results in terms of reducing burden and increasing perceived support and positive gains [79]. Moreover, of note is the iCare Stress Managing e-Training Program [80], which contains educational materials and short videos demonstrating less and more effective ways to handle stressful caregiving situations (accessible through www.icarefamily.com). Though caregivers reported less stress compared with those receiving an education-only online program (while depressive symptoms reduced similarly in both groups), a 30% dropout rate was higher than anticipated. Web-based programs have the potential of reaching more caregivers in need, especially younger ones who are more familiar with the internet. However, methods to enhance participant retention and adherence to such programs need to be explored in the future.

Last but not the least, Au and colleagues [29, 30] found, in a telephone-delivered psychoeducational program, that psychoeducational strategies combined with behavioral activation (to increase positive activities) and communication skills reduced depressive symptoms in Hong Kong Chinese caregivers to a greater extent than psychoeducation alone. This study was novel not just because of the use of telephone but also because it used trained volunteers to deliver the program which clearly was a less costly approach.

### Translational Programs

Taking these kinds of programs another step forward are translational studies, such as CarePRO (Care Partners Reaching Out) [81] and Reaching Out — San Diego [57]. In the former, caregivers are taught direct and indirect self-care strategies similar to those included in other psychoeducation studies. After training staff at local chapters of the Alzheimer’s Association in Arizona and Nevada, this program has been implemented widely in these states with excellent outcomes in terms of reduction of depressive symptoms and perceived burden. Reaching Out — San Diego involved adapting the CWC program and the REACH II program (to be described below) for a primarily Latino clientele in southern CA. As with CarePRO, this program was implemented by a community-based organization, the Southern Caregiver Resource Center, that recruited community volunteers (referred to as Promotoras) to assist in leading the programs, with similar excellent outcomes for depressive symptoms and caregiver burden. Both programs are now sustained by the agencies involved. Such organizational efforts are essential for achieving longer-term outcomes [82]. Similar translational programs are in progress for behavior management skill training, such as the STAR-C in the state of Oregon [83]. Generally, in such programs, emphasis is placed on helping caregivers develop highly structured technical skills to deal with specific problem behaviors by CRs.

It is noteworthy that the US Administration on Aging (now called Administration for Community Living) awarded multiple grants to states wanting to develop shorter, less expensive versions of REACH II and many successful adaptations were made. For instance, the recent development of a four-session approach that includes home visits and telephone support but in smaller doses has been reported [84]. The website of the Rosalynn Carter Institute (www.rosalynncarter.org) provides links to a database that rates interventions based on the quality of research evidence and whether they are “implementation ready” (i.e., with readily available training materials). Here a number of REACH II spin-off programs can be found.

### Therapeutic Mechanisms

Despite the impressive array of intervention studies, it is interesting to note that not many have examined the mechanism of change involved in the therapeutic change. This is certainly an area that needs to be developed in future research. One of the earliest attempts to identify such a mechanism noted that effective utilization of the skills taught mediated the effect of CWC on outcomes, and there was no indication that any skill domain was more important than the others [36]. In contrast, in the START (modeled after CWC) trial, emotion-focused coping mediated the treatment outcome only for those with high depressive and anxiety symptoms at baseline; no effect was found for other coping strategies [85]. In another study, reduced dysfunctional thoughts and increased leisure activities were found to mediate the effect of CBT on depressive symptoms [17]. Furthermore, in two RCTs on variants of the benefit-finding intervention, Cheng and colleagues [19, 20] found consistently that the treatment effects were partially mediated by the increase in self-efficacy in controlling upsetting thoughts during treatment, but not self-efficacy in other domains; a mediating but inconsistent effect of positive gains was also found. Because of the paucity of studies, it is difficult to say whether these therapeutic mechanisms would be generalizable across similar intervention studies.

## Cultural Issues in Dementia Caregiver Interventions

With rising international migration, having the skills to deal with the diverse needs of a multiethnic clientele is becoming more important than ever. However, adapting interventions to the cultural context can be quite challenging [57, 86]. Variations in attributions about dementia have been noted in the literature. For instance, dementia may be conceptualized as part of normal aging or even as retribution of previous wrong-doing in some non-European cultures [8]. Uppal and Borecenas [87] noted the tendency with some South Asian groups to understand dementia as a consequence of family conflict or lack of family support. The most appropriate course of action, according to this understanding, is to resolve the issue within the family. On the other hand, African Americans, due in part to access to extended church and family support networks, tend to rely more on prayer and reframing difficult circumstances [88]. Dementia may also be attributed to unfriendly spiritual forces [89]. Cultural factors may influence how dementia is being recognized and the subsequent decision to seek help and to adhere to treatment recommendations. More research in different cultural contexts is needed to enable a deeper understanding of cultural issues in caregiving and to ascertain the cross-cultural applicability of intervention methods. In order to promote cross-cultural research, instruments would need to be translated and validated in different languages [90], preferably with cross-cultural equivalence established.

A related issue is intervention in multicultural communities. Dementia caregivers from ethnic minority groups face additional cultural and systemic barriers. Sayegh and Knight [91] noted that minority older adults with dementia tended to have greater diagnostic delays and, as a result, higher levels of cognitive impairment and behavioral symptoms at the time of diagnosis. Lower levels of acculturation and lack of knowledge about dementia and health systems are some of the risk factors of delayed help-seeking among ethnic minorities. Furthermore, there is also a lack of concordance in communication and expectation between minority service recipients and health professionals who do not have sufficient knowledge of the language and culture of the ethnic group [92]. In developing countries, there is also a substantial delay in diagnosis and treatment, but it is usually related to lack of resources and lack of training capacity to produce more professional workers, which in return is related to the traditional reliance on the family to provide support in many societies [93]. Furthermore, intervention content may need to be adapted to culturally salient issues as over-devotion and guilt may be more salient to cultures strong in familism.

Additionally, to improve accessibility, acceptability and utilization of services and interventions, language and cultural barriers need to be addressed, and public stigma about dementia needs to be tackled through community education. Interventions and their methods of delivery have to be adapted in light of the resources of the community. Other potential ways to reach out to caregivers are the use of new information/communication technologies and volunteers or paraprofessionals (especially ex-caregivers) [57, 86]. At this time, there are no studies that directly compare traditional interventions with technology-delivered ones, and so we do not yet know if effects are similar. But more important is analysis of the relative cost-effectiveness of the different interventions and modalities of delivery, as policy and clinical decisions will not be based on effect sizes alone. Additional translational research studies will no doubt shed more light on the advantages and disadvantages of any given intervention or combination thereof, in terms of their feasibility and cost-effectiveness.

## Discussion

This review examines the status of the literature on dementia caregiver interventions, with a focus on evidence-based interventions that are based on psychological perspectives and methods. Research has suggested the importance of going beyond the focus of managing objective stressors such as neuropsychiatric symptoms. This general model based on the situation of the CR is overly simplistic, as it ignores caregivers’ characteristics as contributors to their own distress. A host of factors that concern how caregivers interpret these stimuli and respond to them consequently are equally in need of attention. Dysfunctional thoughts, activity restriction, and experiential avoidance are some of the factors informed by theories of psychopathology that have been found to be significantly associated with caregiver health and well-being [16, 25, 28]. These theoretical models have also guided intervention.

As has been mentioned, psychological approaches based on the cognitive-behavioral paradigm has been applied to provide support to caregivers for two decades and has received the most consistent support in the literature. Established methods such as CBT [7, 8] and behavioral activation [30] have been found to yield generally moderate to large effects on caregiver distress such as depressive symptoms. Other approaches derived from CBT principles, such as benefit-finding or alternative thinking [19, 20, 44, 47], have also been found to yield impressive results.

Because CBT and related techniques can be delivered in a structured manner, they can be readily incorporated into psychoeducational programs. This was pioneered by Gallagher-Thompson and colleagues in their CWC program [31]. Since then, a rising trend in the literature is the incorporation of CBT-based techniques into psychoeducational programs, so much so that we believe many of these interventions should be branded as psychoeducation with psychotherapeutic components. Likewise, it is difficult to imagine CBT programs that do not involve educating patients on dementia, community services, and help-seeking, for example. The distinction between psychoeducational and psychotherapeutic programs may be becoming more blurred. An interesting development is that both CBT and psychoeducational programs with psychotherapeutic components have found new ways of delivery via telephone or the internet to reduce administration costs and to reach more caregivers.

Different from traditional forms of CBT, ACT, with an emphasis on non-reactivity (i.e., getting in touch with one’s inner thoughts and feelings without active attempts to alter them), has received support as an equally viable form of treatment for caregivers [50]. In addition, there is emerging interest in helping caregivers to practice mindfulness or mindfulness-based cognitive/stress-reduction techniques which have been shown to reduce rumination and worry in general adult samples [94] and in reducing depressive and anxiety symptomatology in caregivers [62, 66]. Thus, there are now a variety of evidence-based programs based on psychological and complementary perspectives available for caregivers.

### Methodological Issues and Future Intervention Research

Despite the achievements the field has made in intervention science, a few shortcomings, with implications for future research, should be noted. First, we have limited knowledge of the interventions’ effectiveness beyond the usual outcomes of burden, depression, and quality of life; for example, guilt, ambivalence, or grief has received little attention [95]. Second, relatively few RCTs have reported long-term follow-ups. Longitudinal designs with multiple time points of assessment are needed for determining the long-term outcomes of interventions. Third, not every intervention study has assessed treatment fidelity, which is needed for the trial results, whether concerning treatment effectiveness or therapeutic mechanism, to be interpreted unambiguously. Fourth, the outcome variables were sometimes not matched well with what the intervention is expected to achieve. For instance, burden is included as an outcome variable in virtually all studies but it may not be a target of certain intervention methods such as CBT. Participants should display characteristics suitable for the intervention, such as showing elevated depressive symptoms for a CBT-based intervention. Hence, caregivers need to be assessed prior to an intervention the target variables that are coherent with the underlying theoretical model. Fifth and relatedly, studies need to investigate caregiver characteristics that predict response to treatment. For example, caregivers with higher burden and depressive symptoms, having to deal with more challenging behaviors but less cognitive impairment, and who had a home aid at baseline benefited more from REACH II [96], whereas caregivers who reported longer caregiving hours at baseline were found to benefit more from a behavioral activation intervention [30].

Sixth, while RCT researchers are often focused on whether the treatments impact on the main outcomes, attempts to investigate the underlying therapeutic mechanisms are few. Note that the initial theoretical assumption may turn out to be wrong, as was demonstrated in Cheng and colleagues’ studies [19, 20] showing that it was self-efficacy in controlling upsetting thoughts, rather than positive gains as had been assumed, that primarily mediated the treatment effects. Knowledge about how the treatments work will be needed to refine the treatment protocols; for example, the most efficacious components may be isolated and repackaged into briefer treatments for caregivers who often find a complicated and lengthy intervention a burden on top of their daily duties.

Seventh, another area of under-development is family intervention, despite the fact that family issues are often a major source of stress for caregivers [97]. As was suggested by Gaugler et al.’s study [59], family members may be unwilling to come or the caregiver may not want to involve them. In any case, such intervention studies will likely be limited to family members who are reasonably supportive of the caregiver and/or the CR. It is still not clear how to deal with families in which members are detached or hostile with each other or to incorporate family approaches into existing interventions for caregivers. Clearly, more work needs to be done on strengthening the family as a caregiving unit.

Lastly, also needed are more studies to investigate caregiver interventions for the rarer forms of dementia (e.g., frontotemporal or Lewy body dementia) and how interventions should be tailored to their specific needs [98]. Currently, such information is very limited. Likewise, more work is needed to understand caregiving issues in gay, lesbian, and transgender communities including service needs and barriers, particularly in long-term care settings.

### Clinical Translation

We would like to end this review with some comments on translational issues. Being able to deliver the most relevant, individually tailored intervention(s) to each caregiver is going to be the key question for translating research findings into clinical practice. When considering the array of intervention programs, one might ask which ones are most effective for a specific caregiver at varying stages in the dementing process. At present, we cannot clearly answer that question. One might be tempted to consider effect sizes as a useful index for ranking program efficacy. Generally, there is evidence that multicomponent interventions with structured techniques to develop or improve specific skills tend to have larger effects than those focusing mainly on providing social support. However, it is also because of the complexity of these interventions that unambiguous conclusions about what drive which treatment effects, and whether there are synergistic effects between components, are not possible.

In general, information about the particular dementing illness would be most relevant in the aftermath of receiving the diagnosis. Subsequently, skill training may be more useful when caregivers have to learn how to adapt their behaviors and responses in order to manage the problematic behaviors in their CR. As the CR further deteriorates in the middle and later stages, grief-focused interventions as well as skill training for handling dependencies in activities of daily living may be very relevant. At any point in the process where mental health issues such as depression, anxiety, or anger are present, individual, group, and/or family counseling/psychotherapy or related approaches should be considered. For instance, caregivers with plenty of dysfunctional thoughts would probably benefit from CBT [7, 8] or other thought modification methods [19, 20, 44]. Implicit in the above discussion is the need to develop clinical tools to enable assessment of caregiver needs and priorities for the purpose of tailoring the intervention to the individual.

### Concluding Comments

We reviewed four types of caregiver interventions that have psychological ingredients, namely psychoeducation, counseling and psychotherapy, multicomponent interventions, and mindfulness-based interventions. There is a recent surge of psychoeducation and multi-component programs based on psychotherapeutic principles. Knowledge about how these programs work is just emerging and more research on therapeutic mechanisms is needed so that the active ingredients can be identified and refined. The variety of interventions and delivering media underscore the attempt to address important challenges of translating research into practice in a diverse environment. More work needs to be done to tailor interventions to meet the specific needs of each caregiver and to strengthen the family as a caregiving unit.
